# Russian Students’ Social Representations of Higher Education

**DOI:** 10.3390/bs10010002

**Published:** 2019-12-18

**Authors:** Marianna E. Sachkova, Galina K. Esina

**Affiliations:** 1 Department of Psychology, Russian Presidential Academy of National Economy and Public Administration, 119571 Moscow, Russia; 2 Department of Social Psychology, Moscow State University of Psychology and Education, 127051 Moscow, Russia; g-lady@list.ru

**Keywords:** social representations, higher education, students, distance to object

## Abstract

The ideas of the theory social representations proposed by Moscovici and developed in the structural approach by Abric were used in the research in order to reveal the structure and content of students’ social representations of higher education in a modern society. The total sample size was 572 students: of which 197 were secondary school students (average age of 16.7), 189 were undergraduate students (average age of 20.8) and 186 were master students (average age of 29.3). The methodology of Vergès for the analysis of the structure of social representations was used. We tested the hypothesis that the structure of social representations of higher education has general and specific features correlated to the age and educational level of students. It was found that the social representations of schoolchildren, undergraduate students and master’s students differ in a number of elements and content characteristics. Generally social representations of students with different education levels had similar characteristics. Students of secondary schools expanded the core performance, in contrast to students of universities, presumably because of their fewer social experiences, providing large “distance” to higher education as an “object”.

## 1. Introduction

A higher education is the basis of one’s self-development, a necessary start of career and for further success in the life. The global changes in a socio-political and economic system in Russia, as well as wide educational reforms with multi-stage scheme of high school (bachelor, master, post-graduate) transfer social representation studies into the relevant topic of scientific discussion, mostly in the sphere of higher education, its tasks and issues [1].

The theory of social representations by Moscovici describes social representations via ordered, related images, ideas, knowledge, and beliefs in relation to the changing events, shared by individuals included in different social groups [2,3,4,5,6,7,8,9,10,11]. At the same time, society does not serve only as a source of information, but also as a source of a meaning. Social representations are conscious and play an important role in the regulation and maintenance of interpersonal and intergroup relations in different communities. The main provision of this concept lies beyond the area of human reaction to the objective stimulus of the surrounding reality, but also in the subjective representation. The social representation theory assumes an existence of a dynamic interdependence between the specific, culturally conditioned forms of thinking fixed by means of language, and their transformation at the expense of activity of individuals and groups. It is possible to tell, not only about individual social representations, but also about the collective social representations characterizing separate social groups [1,8,12,13,14].

There are different approaches for the analysis of social representations: the socio-genetic (anthropological) approach, the socio-dynamic approach, the discursive approach, and the structural approach [3,4,12,13,14,15,16,17,18,19]. Some studies offer different research methods related to the structural theory. However, most of works are focused on the social representations theory in general and only few of them are related to the structural approach of social representations [1,3,4,12,13,14,15,16,17,18,19].

The structural approach to study the social representations involves their description through the designation of certain components: the core and the periphery [1,3,4,12,13,14,15,16,17,18,19]. The core includes stereotypes and prototypes associated with the object and is characterized by sufficient resistance to change. It is corresponded to historical development, collective memory, values and norms of the group. The periphery is changeable and reflects the essential features of the social representations’ transformation. An analysis of scientific sources shows that the research in the field of social representations focuses mainly on the relationship of an individual or a group with various social objects. The group’s association with the object of representation seems to be a good resource for the analysis and explanation of the processes related to the development, structuring, functioning and dynamics of social representations. The social experience also plays a major role in a social representations research [4,12,13,14,20].

In a structural model the dynamics of representations is related to the action of a number of socio-cognitive mechanisms represented as changing “levels”. In this dynamic, we can distinguish the transformation process and the conditions that contribute to this transformation. The change of social representations takes place through the social experience that plays a special role as a gradual introduction into the social reality of the objects’ environment [3,4,11,12,13,14,17,19].

Abrik developed the approach to the internal structure of social representations [1,4,5,12,13,14,19]. He also mentioned the “distance to the object”, which could help to clarify the specifics of social representations of any object. The leading factor in the functioning of the central core of social representations is the process of activation. Abrik revealed that some elements of a stable system formed by the central core could be “requested” or “used” in different ways to determine the value of both the object itself and the experience associated with it. The importance of the activated elements was directly connected with the role they play in social representations [4,12,13].

The higher education is connected not only with social and professional realization, but also with the formation of value orientations. It depends on how the youth relates to the process and the result of educational activities [1,17,19,20]. Students generate their social representations through a number of negotiations between their own acquired experience and all the representations, that are mediated through the family, the school, the media [1,19,20]. Some studies of various aspects of the educational process and social representations about it were conducted [17,19,20]. The social representations of career were considered by Paschenko de Previl [17]. Tafani and his colleagues developed the experimental approach to the study of the role of self-esteem in the dynamics of social representations of higher education [19].

Thus, understanding youth expectations about their education and future as a whole investigation is studied in the frame of social representation theory. However, there has not been enough research in Russia about youth representations dealing with educational problems. So, the aim of this study is to identify and describe the structure and content of students’ social representations about higher education.

We formulate the following empirical hypothesis: structure of students’ representations about higher education has differences depending on the age and level of studies. We suppose that it is determined by a distance to a perceived object.

## 2. Methods

### 2.1. Participants

A total of 572 students: 197 secondary school students (average age of 16.7), 189 undergraduate students (average age of 20.8) and 186 master students (average age of 29.3) were involved in the research. Participants were recruited from a variety of educational institutions in Moscow.

### 2.2. Materials and Procedure

The research was implemented from November 2017 to March 2018. All respondents were asked to take part in the research; the purpose was formulated as follows: “We are interested in understanding how you perceive higher education”. The research was conducted by the authors during classes and a session lasted 45 min. The sample was purposeful using intact groups. All respondents were provided with feedback, and the results of the study were presented in a summarized form.

Data were collected via a paper and pencil survey.

The questionnaire was consisted of several parts. The instruction was given in writing.

Three steps were carried out: (a) participants were asked to produce five associations with words “higher education” that came spontaneously to their mind; (b) they asked to rank the five produced words in accordance with their perceived importance; (c) they asked to assign a value from 1 to 10 for each of the five produced words in accordance with their perceived significance.

Socio-demographic questions were also asked.

### 2.3. Data Analyses

We used Vergès’ methodology for the analysis of the structure of social representations. All students’ associations were assessed by a prototypical analysis with two parameters—(1) the rank of the occurrence of a separate concept; and (2) the frequency of the occurrence of this concept in the respondents’ answers.

The free associations corpus was analyzed using the IramuteQ program. This software program highlights the content of social representations and reveals the organizing elements of this content on the basis of a lexicographical analysis. We used the rank-frequency processing, which, based on the cross-tabulation of the appearance frequency of the term and its mean appearance ranking, was also used in the study [5,12,13]. The named terms were considered as more or less salient on the basis of these criteria. The analysis was carried out on terms, which were indicated by more than 10% of respondents [5,12,13]. The cross-tabulation of rank-frequency processing allows us to produce a four-cell table which represents the four distinct zones of the representations (see Table 1). For the rank-frequency processing: (1) cell one contains the elements which have a high possibility of forming the “organizing core” of the social representation (mentioned first and frequently); (2) cell four contains the terms of the periphery (mentioned last and the least frequently); (3) cells two (with high frequency and high ranking) and three (mentioned first with low frequency) constitute the potential change zones [5,12,13].

### 2.4. Statistical Methods

The Mann-Whitney U-test of statistical analysis was applied.

## 3. Results

A total of 985 words were evocated by secondary school students in case of stimulus “higher education”. The statistical analysis was carried out on words, which were indicated by more than 10% of respondents [5,12,13]. Thus, 528 words were analyzed. The rank-frequency processing of the corpus demonstrated that the terms “income”, “profession”, “studies”, “exams” and “university” formed part of the organizing core (cell one) (see Table 2). The terms “success”, “labor” (cell three), and “job”, “knowledge” (cell two) were found in the potential change zone. Moreover, the terms “acquaintances”, “future”, “capabilities”, “development”, “responsibility” and “status” were found in the periphery (cell four).

The most frequently mentioned core element is “income” (56 respondents), which has the significance 7.6 and emotional rating 5.89. Another core element is “profession”, which has the highest emotional rating (6.06). These both elements are related to the completion of higher education and specialization in a certain professional field. The one of the infrequently core element “university” (37 respondents) has the highest significance (8.51). Another core element—“exams” has higher significance (8.23) and low emotional rating (3.28).

A total of 945 words were evocated by undergraduate students in case of stimulus “higher education”. The statistical analysis was carried out on words, which were indicated by more than 10% of respondents [5,12,13]. Thus, 462 words were analyzed. The rank-frequency processing of the corpus demonstrated that the terms “job”, “knowledge” and “diploma” formed part of the organizing core (cell one) (see Table 3). The terms “university”, “status”, “prestige” (cell three), and “income” (cell two) were found in the potential change zone. The terms “profession”, “development”, “future”, “erudition” and “studies” were found in the periphery (cell four).

The most frequently mentioned core element is “job” (67 respondents), which has the significance 6.62 and emotional rating 5.89. The core element “diploma” has the highest significance (8.54) and low emotional rating (5.62). Another core element is “knowledge”, which has the highest emotional rating (6.23).

A total of 930 words were evocated by master students in case of stimulus “higher education”. The statistical analysis was carried out on words, which were indicated by more than 10% of respondents [5,12,13]. Thus, 485 words were analyzed. The rank-frequency processing of the corpus demonstrated that the term “knowledge” formed part of the organizing core (cell one) (see Table 4). The terms “qualification”, “university”, “exams”, “intelligence”, “erudition”, “labor”, “career” (cell three), and “diploma”, “development”, “job”, “profession” (cell two) were found in the potential change zone. The terms “studies”, “interesting”, “prestige” and “status” were found in the periphery (cell four).

The single core element “knowledge” has the highest significance (8.43) and positive emotional rating (6.17).

Mann-Whitney U-test was carried out for the following terms: “knowledge” (cell one), “studies”, “status”, “future”, “development” (cell four), “university”, “labor” (cell three). Only two results were significant. The terms from the cell three: “university” (U = 246; *p* = 0.012) and “labor” (U = 169.5; *p* = 0.048) were more significant than other associations.

## 4. Discussion

The aim of this study was to identify and describe the structure and content of student’s social representations about higher education. This study confirmed the hypothesis that the structure of student’s representations about higher education had differences dependent on their age and level of studies. The rank-frequency processing helped to identify the prototypical elements of the object studied. It was confirmed that the core of representations of higher education of secondary school, undergraduate and graduate students differed in the number of elements and their content characteristics. It was attributed to the smaller “distance” of undergraduate and master students to higher education as an “object”. We also found that the core element of social representations of “income” was the meaning of significance for secondary school students, while for higher education students the most consistent was the core element of “knowledge”. The potential change zone of social representations of secondary school students included the elements related to the educational process’ formal organization (“success”, “labor”), which was explained by the greater “distance” to higher education as an “object”. The potential change zone of social representations of undergraduate and master students included the element related to the studying in a particular educational institution (“university”), which was explained by the importance for the future career. In that way, we found that social representations of students about higher education could be developed through further professionalization and professional development.

The peripheral system of students’ social representations had many different associations, including the personal characteristics, the time perspective and the certain status position, that reflected the understanding that the current situation in society determines many factors that affect the higher education’s motives. Thus, the structure undergraduate and master students’ social representations of higher education was characterized by greater elaboration and consistency in comparison with the structure secondary school students’ social representations. Our results were similar to the findings by Abric and Dany, who showed that the individuals who had short distance to an object, had a greater knowledge of it, feel more involved with it, and develop more practices related to this object [4].

Undergraduate and master students were similar in categories “job”, “knowledges” and “diploma”. However, two of them were not included in the core of representation of master students. It was noteworthy that students pursued different goals in higher education. For secondary school students a good income was more important as a success rate in the adult world and entering university become current age task. For undergraduate students, the main aim was getting a diploma for a future job. Master students entered the second degree of education, because they wanted to expand the stock of knowledge and professional skills. They had the most agreed views about higher education. This was due to extensive experience and their immersion of the educational process. These results were similar to the findings by Tafani, Bellon, Moliner, who showed the importance accorded to higher education and the value attached to the efforts it required [19]. The difference from our study was that “knowledges”, “culture”, “intellectual enrichment”, “willpower”, “thinking” and “professional future” had become core elements of representations of college students, and “diploma”, “job”, “difficulty” were peripheral components. Russian secondary school students seemed to be more concerned about passing the final exams, as it could influence their admission to the universities and eventually the higher education’s availability. In addition, they suggested that social representations about higher education might depend on historical and socio-ideological situations and strongly related to social values and socio-economical or culture status [19].

## 5. Conclusions

The main objective of the present study was to reveal and to compare lay thinking about higher education in groups of Russian students. Using the structural approach, this study allowed us to develop hypotheses regarding the representational structure associated with higher education. This study is highlighted some elements of stability concerning knowledge associated with higher education in the Russian context [1] but also, more broadly, in the European context [19] (“job”, “knowledge”, “profession”). As a composite object, some aspects of higher education are inscribed in time and social environment. Also, these results provide evidence of the social environment related to higher education via daily exchanges and practices (“studies”, “exams”, “development”, “future”) as well as via different educational institutions (“university”) and educational programs (“qualification”, “diploma”). Therefore, it should be noted that the definition of higher education functions at an international level [19,20]. However, although certain elements may be universal to various sociocultural environments, it seems to us that the specificities of these contexts (“university”) have an impact on representations as well as on the more concrete and material perspectives of higher education (“income”, “career”). It therefore seems appropriate to study social representations associated with higher education in various sociocultural contacts.

The importance of the observed results is revealed through the understanding of the students’ attitude toward higher education and can be used in a variety of educational institutions to improve the efficiency of the educational services they provide. It is possible to formulate recommendations for development the educational institutions’ organization to improve the socio-psychological competence of the subjects of educational field and the future specialists’ training.

Along with that, some limits should be underlined. Although higher education is a collectively developed and shared object, the social affiliation and participation of individuals lead them to develop new special educational programs. In this regard, it could have been relevant to study further the specificities linked to the variables considered (level of education and socio-demographic variables). However, the use of the dichotomization of level of education (secondary school students, undergraduate students, master students) does not allow us to make a particularly detailed analysis of the results as a matter of the numerous realities that these variables represent. Also, this study shows that future research in this area should strive to identify the correct information about data, because it is the most critical step in conducting studies. In particular, these obtained results may differ between urban and rural participants. In further studies, we plan to explore the representations of students and teachers in different types of groups. Such significant factors as intragroup and family status should be taken into account. Systems of social representations can also differ in people of different genders and professional orientations. Undoubtedly, this requires further large-scale research.

## Figures and Tables

**Table 1:** Analyses of rank-frequency method (as defined by Vergès).

Frequency	Rank	
	Low	High
**High**	Cell 1 “Core zone”	Cell 2 “Potential change zone”
**Low**	Cell 3 “Potential change zone”	Cell 4 “Periphery”

**Table 2:** The structure of secondary school students’ social representations of higher education (N = 197)—rank-frequency method.

Frequency	Rank	
	Low (≤2.88)	High (>2.88)
**High (≥35)**	Income (56; 2.60) Profession (48; 2.60) Studies (43; 2.40) Exams (42; 2.80) University (37; 2.60)	Job (53; 2.90) Knowledge (41; 2.90)
**Low (<35)**	Success (27; 2.70) Labor (24; 2.60)	Acquaintances (35; 3.70) Future (33; 3.00) Capabilities (24; 2.90) Development (24; 3.40) Responsibility (21; 3.20) Status (20; 3.90) ^1^

^1^ The figures in the brackets indicate the frequency and the average rank in which the terms were named.

**Table 3:** The structure of undergraduate students’ social representations of higher education (N = 189)—rank-frequency method.

Frequency	Rank	
	Low (≤2.82)	High (>2.82)
**High (≥35)**	Job (67; 2.80) Knowledge (60; 2.50) Diploma (48; 2.60)	Income (47; 3.20)
**Low (<35)**	University (32; 2.40) Status (28; 2.70) Prestige (27; 2.40)	Profession (32; 3.10) Development (30; 3.20) Future (25; 2.90) Erudition (23; 2.90) Studies (22; 2.90) Qualification (21; 3.40) ^1^

^1^ The figures in the brackets indicate the frequency and the average rank in which the terms were named.

**Table 4:** The structure of master students’ social representations of higher education (N = 186)—rank-frequency method.

Frequency	Rank	
	Low (≤2.90)	High (>2.90)
**High (≥30)**	Knowledge (64; 2.80)	Diploma (47; 3.30) Development (45; 3.10) Job (42; 3.00) Profession (39; 2.90)
**Low (<30)**	Qualification (30; 2.40) University (24; 2.20) Exams (24; 2.80) Intelligence (23; 2.30) Erudition (21; 2.80) Labor (21; 2.80) Career (19; 2.80)	Studies (24; 2.90) Interesting (22; 3.40) Prestige (20; 3.00) Status (20; 3.60) ^1^

^1^ The figures in the brackets indicate the frequency and the average rank in which the terms were named.
